# Brain Response to a Humanoid Robot in Areas Implicated in the Perception of Human Emotional Gestures

**DOI:** 10.1371/journal.pone.0011577

**Published:** 2010-07-21

**Authors:** Thierry Chaminade, Massimiliano Zecca, Sarah-Jayne Blakemore, Atsuo Takanishi, Chris D. Frith, Silvestro Micera, Paolo Dario, Giacomo Rizzolatti, Vittorio Gallese, Maria Alessandra Umiltà

**Affiliations:** 1 Wellcome Trust Centre for Neuroimaging, University College London, London, United Kingdom; 2 Mediterranean Institute for Cognitive Neuroscience (INCM), Aix-Marseille University – CNRS, Marseille, France; 3 Institute for Biomedical Engineering, Consolidated Research Institute for Advanced Science and Medical Care (ASMeW), Waseda University, Tokyo, Japan; 4 Humanoid Robotics Institute (HRI), Waseda University, Tokyo, Japan; 5 Italy-Japan Joint Laboratory on Humanoid and Personal Robotics “RoboCasa”, Tokyo, Japan; 6 University College London Institute of Cognitive Neuroscience, University College London, London, United Kingdom; 7 Department of Modern Mechanical Engineering, Waseda University, Tokyo, Japan; 8 Center of Functionally Integrative Neuroscience (CFIN), Aarhus University Hospital, Århus, Denmark; 9 Advanced Robotics Technology and Systems Laboratory (ARTS Lab), Scuola Superiore Sant'Anna, Pisa, Italy; 10 Neuroprosthesis Control Group, Institute for Automation, Swiss Federal Institute of Technology Zurich (ETHZ), Zurich, Switzerland; 11 Dipartimento di Neuroscienze, Sezione di Fisiologia, Università di Parma, Parma, Italy; 12 Italian Institute of Technology (IIT), Brain Center for Social and Motor Cognition, Parma, Italy; Kyushu University, Japan

## Abstract

**Background:**

The humanoid robot WE4-RII was designed to express human emotions in order to improve human-robot interaction. We can read the emotions depicted in its gestures, yet might utilize different neural processes than those used for reading the emotions in human agents.

**Methodology:**

Here, fMRI was used to assess how brain areas activated by the perception of human basic emotions (facial expression of Anger, Joy, Disgust) and silent speech respond to a humanoid robot impersonating the same emotions, while participants were instructed to attend either to the emotion or to the motion depicted.

**Principal Findings:**

Increased responses to robot compared to human stimuli in the occipital and posterior temporal cortices suggest additional visual processing when perceiving a mechanical anthropomorphic agent. In contrast, activity in cortical areas endowed with mirror properties, like left Broca's area for the perception of speech, and in the processing of emotions like the left anterior insula for the perception of disgust and the orbitofrontal cortex for the perception of anger, is reduced for robot stimuli, suggesting lesser resonance with the mechanical agent. Finally, instructions to explicitly attend to the emotion significantly increased response to robot, but not human facial expressions in the anterior part of the left inferior frontal gyrus, a neural marker of motor resonance.

**Conclusions:**

Motor resonance towards a humanoid robot, but not a human, display of facial emotion is increased when attention is directed towards judging emotions.

**Significance:**

Artificial agents can be used to assess how factors like anthropomorphism affect neural response to the perception of human actions.

## Introduction

Most industrialized countries are aging fast due to an increase of life expectancy and a reduction of child birth rate [1]. In this aging society, it is expected that there will be a growing need for home, medical and nursing care services [2]. For this purpose, robots, and in particular robots with appearance based on the human body, are expected to perform human tasks such as provide personal assistance, social care for the elderly or cognitive therapy [3], and be used in entertainment and education. Just as over the last 30 years the computer business has become an integral part of our daily life, so is robotic technology expected to follow a similar development in the near future [4].

These prospects bring into consideration issues related to natural social interactions with these artificial agents. To become part of our everyday environment, personal robots need to be capable of smooth and natural interactions with humans. It has been proposed [5] that consumer product humanoids should be designed to balance human-ness (to facilitate social interaction) and robot-ness (to avoid false expectations about the robots' abilities). Already several robots have been developed to investigate the socio-emotional aspects of human-robot interactions: animaloid robots like the therapeutic robot PARO [6] and SONY AIBO [7] elicit emotional attachment; humanoid robots like Honda ASIMO [8] and Kawada HRP-2 [9] cooperate with humans; android robots like Actroid [10] and Geminoid [11] explore face-to-face interactions.

The humanoid robot WE4-RII (Waseda Eye No.4 Refined II) was designed to expresses human-like emotions [12] in order to improve the social competence of human-robot interactions [13]. The current study was designed to assess how the neural substrates involved in the perception of human emotions respond to the same gestures impersonated by this anthropomorphic yet clearly mechanical robot, in an endeavour to describe how the agent's appearance modulates brain responses to the perception of emotional facial actions. This research is theoretically grounded in the hypothesis that resonance is pivotal in natural human social interactions [14], [15], [16]. Resonance describes the mechanism by which the neural substrates involved in the internal representation of actions, as well as emotions and sensations, are also recruited when perceiving another individual experiencing the same action, emotion or sensation. While this hypothesis can be traced back as far as William James [17], its interest has been renewed by the discovery of ‘mirror neurons’ in the ventral premotor cortex of the macaque monkey [18], [19]. Mirror neurons fire both when monkeys perform a goal-directed action and when they perceive (see or hear) or infer the same action performed by an experimenter [18], [20]. Neuroimaging studies have identified brain regions, in premotor and parietal cortices [21], [22], [23], in which action execution and observation overlap in the human brain (for review see [24]). The ventral premotor cortex, in particular, constitutes a major locus of motor resonance in humans [24]. Furthermore, the somatosensory cortex responds to the observation and feeling of touch [25], [26], [27], and the insula responds to the observation and feeling of disgust [28]. These examples support a generalization of resonance to multiple domains of cognition including emotions [29], [30].

Artificial agents such as the humanoid robot used in this experiment can participate to a better understanding of factors affecting this resonance, and in particular the role of anthropomorphism. Neuroimaging experiments comparing the observation of humans to artificial agents have yielded mixed results in the inferior premotor and posterior parietal regions of the human motor resonance mechanism. In a PET study, the left ventral premotor activity found in previous experiments of action observation responded to human, but not robot, actions [31]. However, a more recent fMRI study indicated that motor resonance is elicited by a robotic arm and hand [32]. While activity in a neural marker of motor resonance was not significantly related to the anthropomorphism of computer-animated avatars, it decreased with the bias to perceive their actions as biological [33], raising questions about the interaction between perceptual processes related to anthropomorphism, and subjective perception of artificial agents' actions as natural. To address this question, we investigated whether facial emotions expressed by a humanoid robot activate brain regions involved in the perception of human emotions, in particular those engaged in motor and emotional resonance. We used the humanoid robot WE-4RII (Waseda Eye No.4 Refined II), developed by Takanishi Laboratory at Waseda, to express emotions by using facial expressions and the movement of the upper-half of the body including neck, shoulders, trunk, waist, as well as arms and hands [12], [34]. Short videos of the humanoid robot and human actors expressing three emotions (Joy, Anger, Disgust), and silent speech were presented to participants, who were asked to rate either the emotional content or the motion, in order to orient their attention either explicitly to the mental state conveyed by the gesture, or to a purely visual feature, thus privileging an implicit processing of the intentional gesture. On the basis of the mechanical appearance of the anthropomorphic robot, we hypothesized a reduced activity in brain regions involved in motor (ventral premotor and inferior frontal gyrus) and emotional (in particular amygdala and insula) resonance during the observation of the robotic agent compared with the observation of a human agent.

## Methods

### Participants

13 right-handed participants (4 males; aged 29.4+/−7 years) with no history of neurological disorder and normal or corrected-to-normal vision gave their informed consent in writing to take part in this experiment. The study was approved by UCL National Hospital for Neurology and Neurosurgery and Institute of Neurology joint Ethics Committee.

### Stimuli

The humanoid robot used in this experiment, WE-4RII, has 59 degrees of freedom (DOFs), 26 of which are specifically used for controlling facial expression (eyebrows: 8; eyelids: 6; eyes: 3; lips: 4; jaw: 1; neck: 4). A subset of the facial Action Units (AU, described in [35]) was chosen for a simplified but realistic impersonation of the facial gestures used in the experiment - Eyebrows: AU 1, 2, 4; eyelids: AU 7, 42, 43; eyes: AU 5, 6, 43, 44, 45, 46; Mouth: AU 15, 17, 20, 25, 27; Lips: AU 12, 15, 16, 20, 23 [12]. The shoulders have 3 DOFs, plus 2 additional DOFs used for squaring or shrugging gestures. Both the posture and the motion velocity are controlled to realize an effective execution of each gesture.

**Figure 1:**
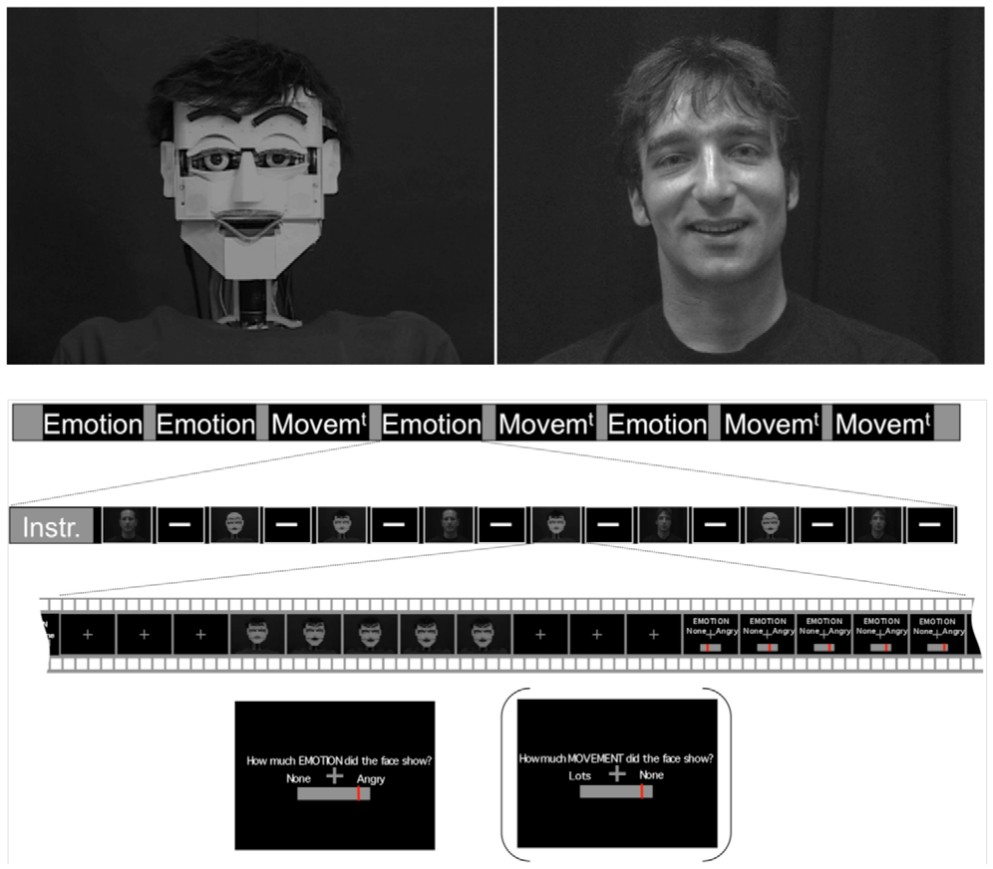
Experimental paradigm. Top: single frame from a Human (left) and Robot (right) Joy stimulus. Middle: organization of an fMRI recording session, showing first, the randomization of the order of the rating blocks (Emotion and Movement) within an acquisition run, then the organization a block starting with a reminder of the instruction (Instr.), and finally the presentation of one stimulus followed by the response screen. Bottom: response screen used in the emotion task (and the motion task between parentheses).

Stimuli consist of 1.5-second greyscale video clips (38 frames at 25 frames per second) showing the agent face and upper body starting from a neutral pose and depicting one of the following gestures: expression of Joy, of Anger, of Disgust and silent Speech. Two different actors were recorded for human stimuli while two versions of the humanoid robot were obtained by the addition of a wig, and four different versions of each type of stimulus were prepared, leading to a total of 64 different stimuli (4 gestures, 2 agents, 2 versions of each agent, 4 versions of each type of stimulus). The greyscale was digitally modified to match the background luminosity and the overall contrast between the human and robot stimuli (see Figure 1, top). Great care was taken to match the dynamics of the human and robot stimuli pairwise (see Video S1).

### Experimental paradigm

There was a total of 16 experimental conditions: across the eight types of stimuli defined by four gestures (Joy, Anger, Disgust and Speech) impersonated by two agents (Human, Robot), participants to the experiment were asked, after each stimulus, to rate the emotional content (“How much EMOTION did the face show?”) or the amount of motion in the stimuli (“How much MOVEMENT did the face show?”).

Participants underwent four sessions of fMRI scanning. Each session contained eight blocks, four in which emotion was rated and four in which motion was rated, presented in a fully randomized order. Participants were informed of the object they rated by a one-word description presented for 1.5 second at the onset of each block (“EMOTION” or “MOVEMENT”, see Figure 1). There were eight stimuli presented in each block in a pseudorandomized order so that each stimulus was seen once in each session and twice for each rating over the course of the experiment. Inter-stimuli onsets were jittered based on a normal distribution of mean 4.5 (+/− SD 0.5) seconds. After each stimulus, the participant's rating was recorded using an analogue scale that ranged from “None” to the target emotion (e.g. “Anger”) to rate emotion, and from “None” to “A lot” to rate motion. The direction of the scale was assigned randomly, and at the onset the response bar was located close to the centre of the scale; the participants pressed a left or right key on their keypad to move the response bar towards the left or the right respectively, and released the key when the response bar reached the desired rate. These characteristics were selected to avoid motor preparation of the response prior to the appearance of the response screen. The duration of the response screen was 1.5 seconds. Prior to scanning subjects were trained with a limited subset of stimuli (3 blocks of 3 stimuli) outside the scanner to become acquainted with the response procedure. Presentation of stimuli and recording of participants' responses were carried out using Cogent (http://www.vislab.ucl.ac.uk/CogentGraphics/index.html) running in Matlab 6.5 (MathWorks™) and analysis of ratings using the statistical program SPSS (SPSS Inc.)

### fMRI data acquisition

Scanning was performed using a 1.5T Siemens Sonata MRI scanner. High-resolution anatomical images were acquired using a T1-weighted 3D MPRAGE sequence. In each of the four experimental sessions, T2*-weighted, gradient-echo, echo-planar imaging sequence was used to acquire 116 volumes containing 48 slices (2 mm thickness and 1 mm gap) covering the whole brain and cerebellum with an in-plane resolution of 3×3 mm (64×64 matrix, fov 192×192×144 mm^3^). The sequence was optimized for blood-oxygen-level dependent signal sensitivity in the ventral cortical areas (orbitofrontal, inferotemporal and amygdala regions) by the use of a tilt angle of −30 degrees and negative phase encoding [36]. The first 4 volumes of each time-series were discarded prior to the analysis to allow for T1 equilibrium. Field maps were also acquired to correct for geometric distortions in EPI images caused by magnetic field inhomogeneities [37].

### fMRI data analysis

fMRI data were analyzed using SPM5 (http://www.fil.ion.ucl.ac.uk/spm), running in Matlab 6.5 (MathWorks™). Slice timing correction was applied to correct for offsets of slice acquisition. EPI volumes were realigned to the first volume for each subject to correct for interscan movement, and unwarped for static magnetic field inhomogeneities using field maps [37] and for movement-induced inhomogeneities using realignment parameters [38]. The high-resolution structural image was co-registered with the mean image of the EPI series, and stereotactically normalised to the Montreal Neurological Institute (MNI) template using sinc interpolation. The normalisation parameters were applied to the EPI time-series, achieving an anatomically informed normalisation. EPI volumes were finally smoothed using an 8mm isometric Gaussian kernel to account for residual inter-subject differences in functional anatomy [39].

The analysis of the functional imaging data entailed the creation of statistical parametric maps representing a statistical assessment of hypothesized condition-specific effects [40]. A random effects procedure was adopted for data analysis. The 1.5-second response periods, and, separately for each of the 16 experimental conditions, the 1.5-second stimulus periods, were modelled at the subject level. These condition-specific effects were estimated with the General Linear Model, with each condition being defined with a boxcar function convolved with the canonical hemodynamic response function. Low-frequency sine and cosine waves modelled and removed subject-specific low-frequency drifts in signal, and global changes in activity were removed by proportional scaling. Each component of the model served as a regressor in a multiple regression analysis.

The brain response to the human stimuli irrespective of the gesture was investigated by contrasting human stimuli presentation, across the four gestures and the two ratings, against the global mean. The resulting statistical maps were entered in a second-level one-sample *t*-test. Similarly, brain response to the human stimuli for each gesture was investigated by contrasting human stimuli presentation, for each gesture and across the two ratings, against the global mean, and entering these contrasts in four second-level one-sample t-tests. All contrasts were thresholded at *p*<0.05 FDR-corrected with an extent threshold of 20 voxels. Anatomical localization was performed using a brain atlas [41] and, when possible, statistical localization relied on probabilistic cytoarchitectonic maps [42]. Other functional attributions relied on comparisons with the literature.

To address specifically the scientific hypothesis, regions responding to the perception of human gestures were further explored to assess their response to robot gestures using a Region Of Interest (ROI) approach. The SPM extension toolbox MarsBar (http://marsbar.sourceforge.net/) was used to extract percentage signal change in 5-mm radius spherical ROI centred on the maximum of the cluster under investigation. Percent signal changes were further analyzed using ANOVA and *t*-tests implemented in the statistical program SPSS (SPSS Inc.), with a significance threshold of 0.05. Regressions (reported at *p*<0.05) between percent signal change and emotional ratings of robot and human stimuli were assessed in brain areas responding specifically to single gestures.

## Results

### Behavioural data

It was shown in a separate experiment [12], and confirmed in preliminary tests with the stimuli used in the present experiment [43], that the robot depictions of the three emotions used in this experiment (Anger, Joy and Disgust) were correctly recognized above chance levels (all >75% correct recognition).

**Figure 2:**
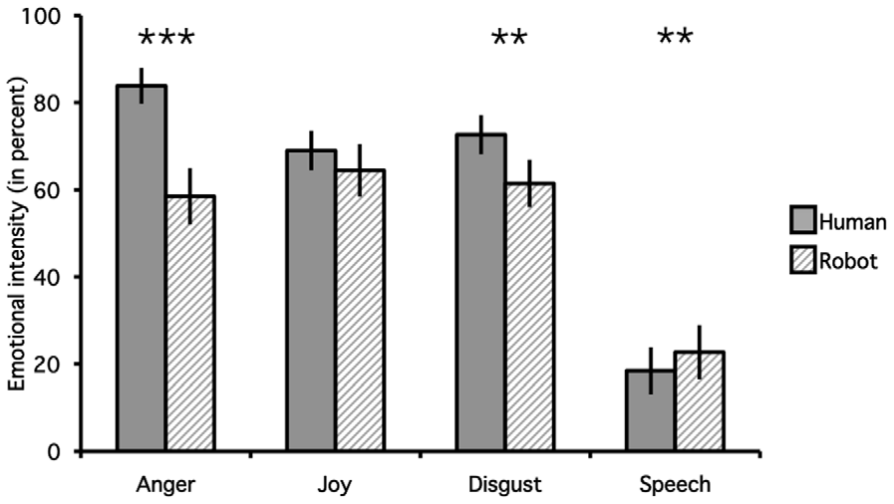
Emotional ratings. Mean (error bar: standard error of the mean SEM) of the percentage ratings of emotional intensity for the four types of gestures depicted by Human (plain color) and Robot (stripes) agents. Emotional ratings are significantly higher for the human in the case of Anger (***: *p*<0.001) and of Disgust (**: *p*<0.05) and for the robot in the Speech condition (**: *p*<0.05).

Repeated-measures ANOVA indicated a significant effect of the Agent (F_1,12_ = 16.1; *p* = 0.002) and the Gesture (F_3,36_ = 57.0; *p*<0.001) on the emotional ratings recorded during the fMRI experiment, as well as a significant interaction between the two factors (F_3,36_ = 12.2; *p*<0.001). As expected given the lack of emotions for the gesture Speech, contrasts revealed significantly increased ratings for Joy, Disgust and Anger compared to Speech (*p*<0.001) irrespective of the agent (see Figure 2). Repeated-measures ANOVAs assessed the effect of Agent on subjects' emotional rating for each gesture separately. Their results indicated significantly higher ratings for human than for robot videos for Anger (F_1,12_ = 31.0, *p*<0.001) and Disgust (F_1,12_ = 7.8, *p* = 0.02, see Figure 2). Speech was rated as significantly more emotional (i.e. less neutral) for the Robot than the Human videos (F_1,12_ = 14.7, *p* = 0.003). Differences between ratings of Joy expressed by Human and Robot were not significant (F_1,12_ = 1.4, *p* = 0.262).

### fMRI data

#### Main effect of human stimulus presentation

The main effect of watching human visual stimuli against the global mean irrespective of the gesture and independent of the rating, yielded bilateral activity in occipital, temporal, parietal and frontal cortices (Table 1). A large cluster (#1, k = 4001 voxels) extended from extrastriate cortices to ventral and lateral temporal cortices bilaterally and to the inferior parietal lobule in the right hemisphere. Extrastriate maxima were attributed to Brodmann areas 17 and 18 bilaterally as well as to the right hemisphere functional areas V3v, V4 [44] and V5 [45]. In the right temporal cortices, maxima were reported at the junction between the occipital and temporal lobes, a region responding to the perception of faces (MNI coordinates 42, −68, −6, compared to 43, −67, −9 in [46]) referred to as the lateral face area (LFA) hereafter (see also [47]), in the fusiform gyrus at the vicinity of the fusiform face area, or FFA, (MNI coordinates 42, −62, −20 compared to 40, −56, −15 in [48]), and in the posterior superior temporal gyrus (MNI coordinates 58, −36, 10 compared to 50, −34, 4 in [49]). In the left hemisphere, clusters were found in V3v (#2), V4 [44] and V5 [50] (#3), as well as in the left-hemisphere FFA (MNI coordinates −34, −62, −18 compared to −35, −64, −16 in [48]), but not in the lateral temporal cortex.

**Table 1:** Main effect of the human stimuli presentation (p<0.05 FDR-corrected, extend k>20; clusters are ordered by cortical lobes, then decreasing z coordinate).

Location				Statistics					
Anatomical		Functional		Coordinates			Zeq	k	#
Occipital lobe				x	y	z			
Right	Superior occipital gyrus	18	70%	28	−98	12	4.51		#1
Right	Middle occipital gyrus	V5	20%	52	−66	8	4.53		#1
Left	Middle occipital gyrus	V5	50%	−44	−72	4	4.45		#3
Left	Middle occipital gyrus	V3v	30%	−26	−98	0	4.85	344	#2
Left	Cuneus	17	50%	−10	−108	−2	4.73		#2
Right	Inferior occipital gyrus	*LFA*		42	−68	−6	4.78		#1
Right	Inferior occipital gyrus	V4	40%	34	−86	−8	5.10		#1
Right	Lingual gyrus	18	90%	20	−88	−12	4.78		#1
Right	Middle occipital gyrus	V3v	50%	28	−90	−12	4.63		#1
Left	Lingual gyrus	V4	30%	−22	−88	−18	4.79	821	#3

Temporal lobe									
Right	Superior temporal gyrus	*22*		58	−36	10	4.10		#1
Left	Fusiform gyrus	*FFA*		−34	−62	−18	4.46	499	
Right	Fusiform gyrus	*FFA*		42	−60	−20	5.10		#1

Parietal lobe									
Right	Postcentral gyrus	2	80%	48	−36	60	3.26	29	
Left	Superior parietal lobule	7p	40%	−14	−66	60	4.35	170	
Right	Superior parietal lobule	7p	70%	16	−70	58	4.49	178	
Right	Inferior parietal lobule	40	50%	58	−34	20	5.17	4011	#1

Frontal lobe									
Right	Precentral gyrus	6	30%	50	4	44	3.87	245	#4
Left	Pars triangularis	44	10%	−38	4	30	3.43	21	
Right	Pars triangularis	44	10%	40	4	30	3.55		#4
Left	Pars triangularis	45	10%	−38	16	30	4.38	55	
Right	Pars triangularis	45	50%	48	24	26	3.50	192	
Left	Pars triangularis	45	20%	−46	36	10	3.64	20	
Left	Pars triangularis	44	20%	−42	24	2	3.73	110	

When available, functional localization is based on the anatomy toolbox [42], with percentage indicating the probability of the maximum belonging to the designated area. #*i* is used when more than one maximum is reported for cluster *i*.

**Figure 3:**
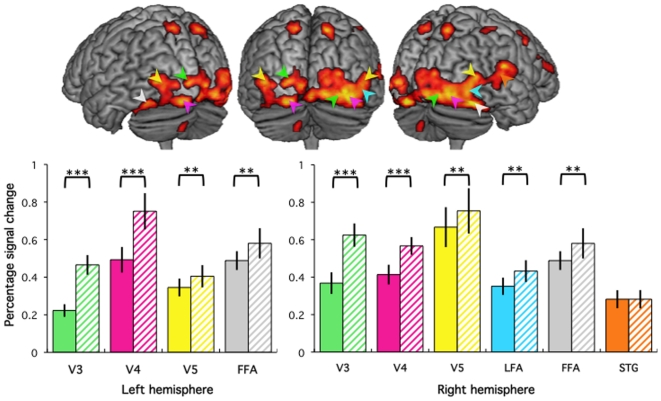
Occipital cortices. Top: Main effect of human stimuli presentation (FDR-corrected *p*<0.05, extend k>20) overlaid on a standard brain, seen from the back (middle), back-left (left) and back-right (right). Bottom: Bar graphs on the left give percent signal change (error bar: SEM) in response to the presentation of Human (plain colour) and Robot (stripes) stimuli irrespective of the task and action depicted. Coloured arrows indicate the position of the maxima (see also Table 1) used to represent the functional areas (see text for details). Brackets indicate whether signal change significantly differs between human and robot stimuli (*** *p*<0.001, ** *p*<0.05, * *p*<0.1).

Extracted signal changes collapsed across the 4 gestures, in 5-mm radius spheres centred on the maxima localized in V3v, V4, V5 and FFA bilaterally as well as in LFA and STS in the right hemisphere were submitted to 2 (Agent) by 2 (Rating) repeated measures ANOVA. Results illustrated in Figure 3 illustrate the significant effect of Agent in all ROI but the STS, corresponding to an increase of the response to Robot compared to Human agents (V3v and V4 bilaterally p<0.001; V5, FFA bilaterally and right LFA p<0.05), without significant effect of the object of Rating (all p>0.05) nor a significant interaction between Agent and the Rating. There were no significant effects of Agent or Rating nor an interaction between Agent and Rating (all p>0.1) in the right STS.

#### Main effect of human stimulus presentation: frontal cortices

**Figure 4:**
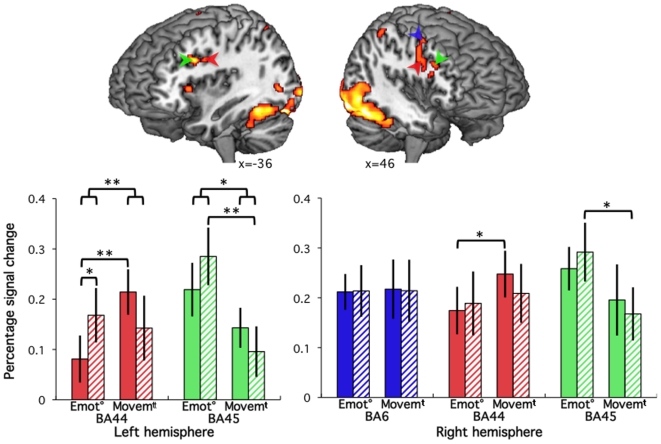
Inferior frontal cortices. Top: Main effect of human stimuli presentation (FDR-corrected *p*<0.05, extend k>20) overlaid on a standard brain, seen from front-left (left) and front-right (right) with cut-outs showing the bilateral inferior frontal gyrii clusters investigated. Bottom: Bar graphs on the left give percent signal change (error bar: SEM) in response to the presentation of Human (plain colour) and Robot (stripes) stimuli during explicit (E) and implicit (I) tasks irrespective of the action depicted. Coloured arrows indicate the position of the maxima used to represent the functional areas (see text for details). Brackets indicate whether significant effects revealed by ANOVAs and paired *t*-test (** *p*<0.05, * *p*<0.1).

Because of our *a priori* hypothesis on the role of inferior frontal cortices in motor resonance, percent signal change was extracted in 5mm radius spheres centred on the maxima of inferior frontal gyrii activated clusters, localized in three Brodmann areas (BA) according to the cytoarchitectonic probabilistic maps [51]: BA 6 in the right hemisphere, and bilateral BAs 44 and 45, located in the vicinity of clusters reported during the perception of a human face performing intransitive mouth gestures [21]. Signal extracted in these ROIs, collapsed across the 4 gestures, was submitted to 2 (Agent) by 2 (Rating) repeated measures ANOVAs (Figure 4). There was no significant main effect or interaction (all *p*>0.5) affecting signal in the right BA6. In the left BA44, there was a significant interaction between Agent and Rating (*p* = 0.02), with no main effect of Agent (*p* = 0.4) or Rating (*p* = 0.8). Paired *t*-tests revealed that response to the robot was not significantly affected by the Rating, while response to human stimuli was significantly increased for the Movement compared to Emotion rating (*p* = 0.04). A similar profile in the right BA44 did not reach significance (all *p*>0.1).

In the left BA45, there is a significant effect of Rating *(p* = 0.05), and a trend in the interaction between Rating and Agent (*p* = 0.06), with no main effect of the Agent (*p* = 0.8). As with BA44, a similar profile in the right hemisphere BA45 did not reach significance (all *p*>0.1). The only significant *t*-test showed that signal change for robot stimuli was significantly increased during rating of the emotional content of the stimulus compared to its motion (left *p* = 0.01, note than on the right *p* = 0.1). The same contrast did not reach significance for human stimuli.

#### Action-specific brain responses

**Figure 5:**
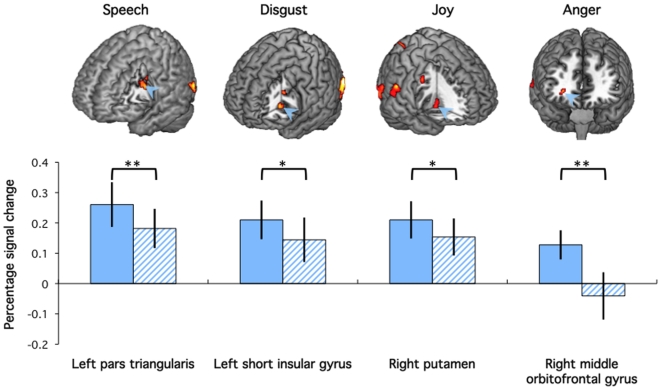
Action-specific responses. Top: Cut-outs showing clusters responding to each type of action (FDR-corrected *p*<0.05, extend k>20) overlaid on a standard brain. Bottom: Bar graphs on the left give percent signal change (error bar: SEM) in response to the presentation of Human (plain colour) and Robot (stripes) stimuli for the corresponding action irrespective of the task. Arrows indicate the position of the maxima used to represent the functional areas (see Table 2 and text for details). Brackets indicate whether significant effects revealed by ANOVAs and paired *t*-test(** *p*<0.05, * *p*<0.1).

Brain response to human stimuli was investigated for the four gestures independently at the second level to isolate brain areas responding to individual gestures (Table S1). Areas responding specifically to each of the four types of facial action against the global mean are provided in Table 2 and illustrated on Figure 5. The left inferior frontal gyrus activity associated with perception of Speech gestures was localized in *Pars Triangularis*, and attributed to Brodmann area 44 [51]. Its location falls into in a subdivision of Broca's region putatively involved in syntactic aspects of speech execution and perception (reviewed in [52]). A similar region was reported for the auditory perception of language coordinates (−46, 12, 24 compared to −40, 14, 28 in [53]). In the present experiment, this area responded to the perception of human speech gestures and was not found in the other types of action, supporting the specificity of its response to language-related actions. Signal change for Speech stimuli extracted in a 5-mm sphere centred at −46, 12, 24 was submitted to 2 (Agent) by 2 (Rating) ANOVA. There is a significant effect of Agent (*p* = 0.05) corresponding to increased signal to human compared to robot stimuli. There was a trend (*p* = 0.09) towards increased response when rating emotion compared to movement.

**Table 2:** Main effect of the human stimuli for one type of action only (p<0.05 FDR-corrected, extend k>20) and used in subsequent investigation.

Anatomical localization		Coordinates			Statistics	
		x	y	z	Z-score	k
**Speech**						
Left	Pars triangularis	−46	12	24	3.80	83
**Disgust**						
Left	Short insular gyrus	−30	22	4	3.90	26
**Anger**						
Right	Middle orbital gyrus	28	40	−4	4.78	24
**Joy**						
Right	Putamen	24	4	−8	3.88	69

See full list of activated areas in Table S1.

The left anterior insula, a mirror region for this emotion (−30, 22, 4 compared to −34, 28, 6 in [28]) was associated with the perception of Disgust gestures. In the ROI associated with this activity, only the main effect of agent showed a trend (*p* = 0.1), corresponding to an increased response to human expressions of disgust compared to robot's expression of the same emotion (paired *t*-test *p* = 0.1). There was no significant effect of the object of Rating, or correlation between emotional rating and activity in this ROI.

The right orbitofrontal cortex was associated with the perception of human expression of Anger. Repeated measure ANOVA indicated a significant main effect of Agent (*p* = 0.01) in the signal extracted in this region, corresponding to an increased response to human compared to robot stimuli. In addition, one-sample *t*-test reveals that response to the robot's expression of anger in this region was not significantly different from the global mean (*p* = 0.3).

Finally, the right putamen, part of the ventral striatum associated with the perception of human gestures of Joy, was the only non-cortical region reported in this section. There was no significant main effect of Agent or Rating on the signal extracted in the putamen, but a trend (*p* = 0.1) towards an increase of response to human compared to robot stimuli. There was a significant correlation between extracted percent signal change during perception of human stimuli of joy and the emotional rating (R^2^ = 0.461, *p* = 0.04), but not for robot stimuli of joy (R^2^ = 0.174, *p* = 0.16). No other correlations between action-specific brain regions and emotional ratings were significant for the human or the robot stimuli.

## Discussion

In the current fMRI study, participants observed short videos depicting emotional (Anger, Joy and Disgust) or emotionally neutral (Speech) facial gestures expressed by real humans or by the robotic humanoid platform WE-4RII, designed to resemble a human face. WE-4RII can reproduce a subset of the facial Action Units [35], by movements of its eyebrows, eyes, eyelids, lips, mouth, neck, shoulder and upper torso, so as to express in a recognizable manner the four gestures used in this experiment [12] while at the same time being perceived as an artificial, i.e. non-human and non-intentional, embodied agent.

Analysis of the ratings of the emotional content by the participants of the current experiment (see Figure 2) indicated that emotional gestures were perceived as more emotional (and the emotionally neutral speech gestures, less emotional) when expressed by the humans than by the robot. The use of stimuli derived from this robotic platform in an fMRI experiment provided a unique opportunity to test whether the reduction of perceived emotionality of the artificial agent is associated with reduced activity in brain areas involved in the feeling or the perception of the same emotions depicted by human agents. Note that because the robot is clearly mechanical compared to human actors, it is not possible to dissociate, in the present experiment, differences in activity related to the appearance and to the artificial nature of the robot. In addition, stimuli were grouped into fMRI blocks during which participants were asked to rate either the emotional content or the movement depicted, as a proxy to orient their attention either towards the intention underlying the gestures (the emotion) or toward a purely visual feature of the stimuli (the amount of movement) so that processing of the mental state causing the action (the emotion being displayed in Joy, Anger and Disgust, the will to communicate in Speech) is implicit [54]. This manipulation was chosen to disentangle bottom-up processes, influenced by the nature of the stimuli, and top-down processes, influenced by the instruction to attend the emotion or the motion of the stimulus [54].

fMRI analysis consisted of, first, isolating regions of interest on the basis of their response to human stimuli, and second, assessing the modulation of their activity by the agent depicting the gestures and by the object of attention. Discussion of the data focuses on regions of the visual association areas in the occipital and temporal cortices involved in the perception of faces and objects; regions found to be specifically associated with the perception of the different types of basic emotions, insula for disgust, putamen for joy and orbitofrontal cortex for anger, and silent speech in the left inferior frontal cortex; and the inferior frontal cortices, which were predicted on the basis of their contribution to motor resonance.

### Visual cortices

Responses to human stimuli are reported in visual areas V3, V4 [44] and V5 [50], and in temporal areas responding to the perception of faces (fusiform face area FFA [48], lateral face area LFA [46]) and actions (superior temporal gyrus, [49]). Activations in these occipital and posterior temporal cortices when perceiving human gestures was predicted on the basis of their essential role in visual perception of biological motion and body parts.

In terms of the effect of robotic stimuli on activity in occipital and posterior temporal visual cortices, the main finding was that all regions, with the notable exception of the superior temporal gyrus cluster, showed an increased response for robot compared with human stimuli. This increase appears at odd with their proposed human face-specificity [55] of FFA bilaterally and right LFA. Already, a bilateral fusiform gyrus activity was reported in response to animal faces depicting actions [56]. Another fMRI study found similar responses when perceiving human faces and animals with or without faces in the same fusiform region [57], suggesting that perception of animals relies on the same substrates of perception of human faces.

Explaining this increased response to the robot's face entails discussing mechanisms involved in the domain-specificity of perception in the FFA. Face perception is holistic [58], and deficits of prosopagnosic patients support that the FFA is crucial for this holistic perception [59]. According to Pinker [60], a perceptual process must be characterized by the type of geometry it pays attention to, and the geometry the human face recognition system is sensitive to can be demonstrated in newborns [61]. Pinker argues any object that shares these geometric features, as the robotic face used here, will be automatically processed by the “face module”. This automatic processing might explain activity in the FFA bilaterally and in the right LFA normally activated by human gestures in response to robot stimuli.

It has been proposed that in the FFA, features of the presented face are compared to an average “face template” [55], [62]. Because the robot face was clearly distinguishable from a human face, this comparison could lead to a reduction of signal, as was the case for the perception of animals [57] or of cartoon faces [63]. Alternatively, this comparison could require additional processing of the visual input in order to recognize the robot as a face. This interpretation is supported by the significant increase of response in extrastriate areas V3, V4 and V5, implied in the processing of low-level aspect of visual stimuli such as form, colour and motion. Furthermore, a similar increase of response has been reported in the visual word form area of the ventral occipital cortex when the visual appearance of a written word is degraded, Altogether, increased response to robot compared to human gestures in visual areas implicated in the perception of faces and actions is likely to reflect additional processing of the unfamiliar stimulus [64].

There is no significant difference in responses to robot and human stimuli in the right superior temporal gyrus. The posterior temporal cortex responds to a large range of stimuli. It is particularly respondent to visual depictions of actions across a variety of presentations (full body or body parts actions [65], point-light displays [66], as well as animal actions [56] and scripted geometrical shapes movements [67]). The finding of a similar response to robot and to human stimuli in this region argues in favour of a fully integrated representation of gestures, as both types of stimuli are similar in most respects but the appearance of the agent depicting the gesture.

### Regions responding to only one type of human gesture

Aside the occipital and temporal regions involved in processing all gestures, some brain areas respond only to one of the human gestures used in this experiment. We are particularly interested in regions known to be involved in the processing (either in execution or in perception) of the specific gesture they were found associated with, namely the insula for disgust and Broca's region for speech.

Activity in the left insula was predicted on the basis of its participation in emotional resonance during the perception of disgust gestures [28]. The short insular gyrus cluster associated with the perception of disgust gestures (−30, 22, 4) was in the vicinity of a left anterior insula cluster in which overlap between observation and feeling of disgust has been reported [28]. This region was activated in response to the humanoid robot's expression of disgust in comparison to baseline, and the trend showing a reduction of its response in comparison to human stimuli did not reach significance (*p* = 0.1). This finding demonstrates emotional resonance towards an anthropomorphic robot in the case of disgust gestures.

Perception of human joy was associated with activity in the right putamen, a brain area repeatedly associated with the induction of happy mood (see meta-analysis in [68]). This can be attributed to its role in reward-processing [69] following the suggestion that dopaminergic signalling in these regions is important to elicit internal rewarding response [70]. Such interpretation supports its involvement in the emotional resonance for Joy. As was the case for the insular cluster associated with Disgust, results indicated that there was a trend towards decreased response to robot compared to human stimuli. In addition, the correlation between emotional ratings and brain activity, significant for human stimuli, was not significant in the case of robot stimuli. Altogether, our data support a reduced emotional resonance towards robotic expressions of Joy in the striatal structure, extending the results from Disgust to a non-cortical area.

The involvement of the orbitofrontal cortex in emotions has been demonstrated by lesion studies in humans [71]. The right orbitofrontal region found here has already been shown to respond to angry faces [72]. Activity was significantly larger in the OFC for human than for robot angry gestures, and the response to robot stimuli was not significantly different from the baseline, suggesting that response of this region was limited to human stimuli. An explanation based on the large difference in perceived emotion of the two agents depicting anger (see Figure 2) can be excluded by the absence of significant correlation between orbitofrontal activity and emotional ratings for either agent. An alternative explanation, according to which the orbitofrontal cortex is involved in top-down aspects of emotional evaluation [73] is contradicted by the absence of effect by the manipulation of attention through rating instructions. The absence of significant response to robot stimuli might result from the role of the orbitofrontal cortex in social cognition. Orbitofrontal lesions have been associated with disinhibited social behaviours, putatively by lack of anticipation of their negative outcomes [74]. We suggest that because of its clearly artificial nature, the robot did not elicit a desire for social contact [75] sufficient to be reflected in orbitofrontal activity. Further investigations including socially rewarding interactions with artificial agents, for example interactions with androids [11], will be necessary to confirm this interpretation.

A cluster associated with the perception of human speech only was attributed to Brodmann area 44 [51], a part of Broca's region associated with speech. This activation was similar to clusters reported for auditory [53], visual [76] and visuo-auditory [77] processing of speech. More generally, Broca's region involvement in language production and comprehension [52] supports a role of motor resonance in the domain of speech perception that was hypothesized prior to the discovery of mirror neurons as the “motor theory of speech perception” [78]. Activity in this region was reduced when speech was impersonated by the humanoid robot, compared with human agents, but significantly activated compared to baseline, suggesting robot stimuli elicited reduced motor resonance compared to human stimuli. In contrast to the inferior frontal activities described in the next section, the absence of a significant interaction between Agent and Rating suggests that this reduced activity was caused by the unrealistic appearance of the humanoid robot.

### Inferior frontal cortices

The inferior frontal gyrii and ventral premotor cortices were scrutinized because of their involvement in motor resonance, important for the perception of actions, and by extension of emotions, expressed by facial [79] and body [80] gestures. Five clusters were isolated, in the left lateral premotor cortex (BA 6), and bilaterally in the posterior (BA 44) and anterior (BA45) *pars triangularis* of the inferior frontal gyrus. This region of the cortex, which has been implicated in the perception of human actions [56] and imitation [22], [81], is likely homologous to frontal regions responding to action observation in macaque monkeys [24], [82].

The agent displaying the emotion had no effect on activity in these regions of interest, in keeping with the responses to the observation of human and robot [32], [83] hand actions that have been reported in this region. Both previous studies and in the present experiment, mechanical robot effectors, respectively a “hand” and a “face”, were clearly associated with a bilateral increase of activity in the inferior frontal cortex, with no significant difference in activity between the robotic and human agents. This supports that motor resonance is recruited irrespective of the agent executing the action. Even point light displays of human body motions evoke motor resonance within Broca's region [84]. Mere resemblance of the body shape is thus sufficient to elicit motor resonance: while mirror neurons in monkeys have been reported anecdotally to respond to conspecifics' actions, most of their recordings have been made when monkeys observed human actions; while there is a generic correspondence between the body shapes and degrees of freedom of the two species, the match is not perfect, implying that mirror neurons can generalize across species. Human neuroimaging experiments presenting human, monkey and dog facial movements suggest that even for the less anthropomorphic agent, the dog, motor resonance can be observed provided the action is part of the observer motor repertoire [biting in contrast to barking; 56]. Recent results using robots, including the present data, support that motor resonance generalizes to anthropomorphic artefacts [32], [83].

This conclusion is consistent with behavioural experiments investigating motor resonance, that demonstrated that the observation of humanoid, but not industrial, non anthropomorphic, robotic gestures [85] cause a motor interference effect [86]. In another line of research using hand action imitation, both real and robotic hands had an action priming effect [87].

In both BA44 and BA45 of the left hemisphere, an interaction between the effect of Agent and of Rating was identified, with a main effect of rating in BA45 corresponding to increased response when attention was explicitly directed towards the emotion. BA44 response to the robot was not influenced by the object of attention, while response to human increased when attention was directed towards the gesture's movement compared to its emotion. In contrast, response of the anterior BA45 to human stimuli was not influenced by the direction of attention, but response to robot stimuli was increased when participants were required to rate the emotion of the stimuli, compared to its movements.

Altogether, these results suggest a modulatory influence of task on the activity of both left inferior frontal areas. One interpretation of our results is the preference for representation of actions' intentions in BA45 [24], similar to the response to abstract actions in the more rostral region of macaque monkey's arcuate sulcus [82]. The main effect of rating in the current experiment corroborated BA45's preference for the representation of intentions underlying the depicted gestures when attention is explicitly directed towards emotion. The pattern of activity in BA45 could thus be explained by the interaction between bottom-up and top-down processes. Bottom-up processes of intention understanding could be automatic for human stimuli, and therefore not sensitive to modulation by attention. In contrast, because the system has no prior representation of robots' actions, robot stimuli would not be processed automatically. Response to robot stimuli would be modulated by the object of attention: stimuli would be processed as intentional actions when the task required assessing the emotion, but as artefact movements when the task did not require processing the emotion. The interaction between Task and Agent in BA45 could thus derive from an interaction between bottom-up processes, influenced by the nature of the agent, and top-down processes, depending on the object of attention.

### Conclusion

Using fMRI, we investigated whether regions responding to human basic facial emotions and silent speech were also activated when a humanoid robot impersonated the same gestures. While robot stimuli elicited larger responses in occipital and posterior ·temporal areas, a reverse pattern was observed in regions responding specifically to one type of human gesture only, namely the left inferior frontal cortex for motor resonance in speech perception and insula for emotion resonance in disgust. We suggest that the clearly artificial appearance of the humanoid robot used in this experiment, WE-4RII, together with the limited number of degrees of freedom available in comparison to a real human, precluded high levels of resonance towards this agent's gestures. While none of the subjects had previous experience with an emotional robot, it is possible that experience leading to the establishment of real relationships with a robot could create a sense of social bonding. Further work should investigate the relation between familiarity with robots and the activity of neural markers of motor and emotion resonance. This first study paves the way for further exploration of perception of robotic actions.

## Supporting Information

Table S1 Main effect of the human stimuli presentation (p<0.05 FDR-corrected, extend k>20, clusters are ordered by cortical lobes, then decreasing z coordinate), provided across the four types of actions and for each action independently. When available, functional localization is based on the anatomy toolbox (Eickhoff et al., 2005), with percentage indicating the probability of the maximum belonging to the designated area. Underlining highlights regions described in Table 2.
(0.13 MB DOC)

Video S1 Experimental paradigm for participants in the fMRI experiment (details in main text).
(0.43 MB MP4)
