# Supplementary material for: Brain Response to a Humanoid Robot in Areas Implicated in the Perception of Human Emotional Gestures
Source: PLoS One. 2010 Jul 21;5(7):e11577. doi: 10.1371/journal.pone.0011577 (PMC2908128; doi:10.1371/journal.pone.0011577)
Supplement: Table S1 — Main effect of the human stimuli presentation (p<0.05 FDR-corrected, extend k>20, clusters are ordered by cortical lobes, then decreasing z coordinate), provided across the four types of actions and for each action independently. When available, functional localization is based on the anatomy toolbox (Eickhoff et al., 2005), with percentage indicating the probability of the maximum belonging to the designated area. Underlining highlights regions described in Table 2. (0.13 MB DOC) [file pone.0011577.s001.doc]

- Table S1: Main effect of the human stimuli presentation (p<0.05 FDR-corrected, extend k>20, clusters are ordered by cortical lobes, then decreasing z coordinate), provided across the four types of actions and for each action independently. When available, functional localization is based on the anatomy toolbox (Eickhoff et al., 2005), with percentage indicating the probability of the maximum belonging to the designated area. Underlining highlights regions described in Table 2.

| **Anatomical** | | **Functional** |  | **All stimuli** | | | **Joy** | | | **Anger** | | | **Disgust** | | | **Speech** | | |
| --- | --- | --- | --- | --- | --- | --- | --- | --- | --- | --- | --- | --- | --- | --- | --- | --- | --- | --- |
| **Occipital lobe** | |  |  | **x** | **y** | **z** | **x** | **y** | **z** | **x** | **y** | **z** | **x** | **y** | **z** | **x** | **y** | **z** |
| Right | Superior occipital gyrus | 18 | 70% | 28 | -98 | 12 | 26 | -98 | 10 | 26 | -98 | 12 | 28 | -98 | 10 | 28 | -98 | 12 |
| Right | Middle occipital gyrus | V5 | 30% | 52 | -66 | 8 | 52 | -66 | 8 | 54 | -66 | 10 | 50 | -66 | 6 | 50 | -66 | 6 |
| Left | Middle occipital gyrus | V5 | 50% | -44 | -72 | 4 | -44 | -72 | 4 | -46 | -72 | 4 | -46 | -72 | 2 | -44 | -70 | 6 |
| Left | Middle occipital gyrus | V3v | 30% | -26 | -98 | 0 | -26 | -98 | 0 | -24 | -98 | 2 | -24 | -98 | 2 | -24 | -96 | 0 |
| Left | Cuneus | 17 | 50% | -10 | -108 | -2 | -10 | -108 | -2 | -10 | -108 | -2 | -8 | -108 | 0 | -8 | -106 | -2 |
| Right | Inferior occipital gyrus | *LFA* |  | 42 | -68 | -6 | 44 | -68 | -8 | 44 | -68 | -6 | 42 | -68 | -8 | 42 | -70 | -4 |
| Right | Inferior occipital gyrus | V4 | 40% | 34 | -86 | -8 | 36 | -86 | -10 | 32 | -84 | -6 | 40 | -84 | -14 | 40 | -84 | -16 |
| Right | Lingual gyrus | 18 | 90% | 20 | -88 | -12 | 18 | -90 | -10 | 16 | -90 | -12 | 20 | -88 | -12 | 20 | -88 | -10 |
| Right | Middle occipital gyrus | V3v | 50% | 28 | -90 | -12 | 30 | -90 | -12 | 34 | -88 | -12 | 34 | -86 | -10 |  |  |  |
| Left | Lingual gyrus | V4 | 30% | -22 | -88 | -18 | -18 | -88 | -16 | -20 | -86 | -16 | -24 | -86 | -20 | -22 | -86 | -18 |
|  |  |  |  |  |  |  |  |  |  |  |  |  |  |  |  |  |  |  |
| **Temporal lobe** | |  |  |  |  |  |  |  |  |  |  |  |  |  |  |  |  |  |
| Right | Superior temporal gyrus | *22* |  | 58 | -36 | 10 | 58 | -38 | 8 | 56 | -38 | 8 | 56 | -38 | 8 | 52 | -40 | 6 |
| Right | Fusiform gyrus | *FFA* |  | 42 | -60 | -20 | 40 | -60 | -18 | 40 | -54 | -22 | 42 | -60 | -20 | 44 | -62 | -22 |
| Left | Fusiform gyrus | *FFA* |  | -34 | -62 | -18 | -34 | -60 | -18 | -40 | -56 | -18 | -38 | -50 | -20 | -32 | -64 | -20 |
|  |  |  |  |  |  |  |  |  |  |  |  |  |  |  |  |  |  |  |
| **Parietal lobe** | |  |  |  |  |  |  |  |  |  |  |  |  |  |  |  |  |  |
| Left | Superior parietal lobule | 7p | 40% | -14 | -66 | 60 | -14 | -68 | 58 | -14 | -66 | 60 | -14 | -64 | 60 |  |  |  |
| Right | Superior parietal lobule | 7p | 70% | 16 | -70 | 58 | 18 | -72 | 56 | 14 | -64 | 58 | 20 | -72 | 56 | 14 | -68 | 58 |
| Right | Inferior parietal lobule | 40 | 50% | 58 | -34 | 20 | 60 | -34 | 22 | 58 | -34 | 18 | 62 | -32 | 20 | 68 | -28 | 14 |
| Right | Postcentral gyrus | 2 | 80% | 48 | -36 | 60 |  |  |  | 44 | -40 | 58 |  |  |  |  |  |  |
|  |  |  |  |  |  |  |  |  |  |  |  |  |  |  |  |  |  |  |
| **Frontal lobe** | |  |  |  |  |  |  |  |  |  |  |  |  |  |  |  |  |  |
| Right | Precentral gyrus | 6 | 30% | 50 | 4 | 44 | 50 | 6 | 42 |  |  |  |  |  |  |  |  |  |
| Left | Pars triangularis | 44 | 10% | -38 | 4 | 30 |  |  |  |  |  |  |  |  |  |  |  |  |
| Right | Pars triangularis | 44 | 10% | 40 | 4 | 30 |  |  |  | 40 | 2 | 30 | 42 | 2 | 30 |  |  |  |
| Left | Pars triangularis | 45 | 10% | -38 | 16 | 30 |  |  |  |  |  |  | -34 | 22 | 28 | -38 | 18 | 32 |
| Right | Pars triangularis | 45 | 50% | 48 | 24 | 26 | 48 | 24 | 24 |  |  |  |  |  |  | 54 | 24 | 24 |
| Left | Pars triangularis | 44 | 40% |  |  |  |  |  |  |  |  |  |  |  |  | -46 | 12 | 24 |
| Left | Pars triangularis | 45 | 20% | -46 | 36 | 10 |  |  |  |  |  |  |  |  |  |  |  |  |
| Left | Pars triangularis | 44 | 20% | -42 | 24 | 2 | -42 | 20 | -8 | -38 | 24 | 4 |  |  |  |  |  |  |
| Right | Middle orbital gyrus |  |  |  |  |  |  |  |  | 28 | 40 | -4 |  |  |  |  |  |  |
|  |  |  |  |  |  |  |  |  |  |  |  |  |  |  |  |  |  |  |
| **Insular** | |  |  |  |  |  |  |  |  |  |  |  |  |  |  |  |  |  |
| Left | Short insular gyrus |  |  |  |  |  |  |  |  |  |  |  | -30 | 22 | 4 |  |  |  |
|  |  |  |  |  |  |  |  |  |  |  |  |  |  |  |  |  |  |  |
| **Internal structure** | |  |  |  |  |  |  |  |  |  |  |  |  |  |  |  |  |  |
| Right | Putamen |  |  |  |  |  | 24 | 4 | -8 |  |  |  |  |  |  |  |  |  |
